# The Novel Compound SUL-138 Counteracts Endothelial Cell and Kidney Dysfunction in Sepsis by Preserving Mitochondrial Function

**DOI:** 10.3390/ijms24076330

**Published:** 2023-03-28

**Authors:** Bastiaan S. Star, Elisabeth C. van der Slikke, Azuwerus van Buiten, Robert H. Henning, Hjalmar R. Bouma

**Affiliations:** 1 Department of Clinical Pharmacy and Pharmacology, University Medical Center Groningen, University of Groningen, 9700 RB Groningen, The Netherlands; b.s.star@umcg.nl (B.S.S.);; 2 Department of Internal Medicine, University Medical Center Groningen, University of Groningen, 9700 RB Groningen, The Netherlands

**Keywords:** sepsis, AKI, oxidative stress, metabolism, SUL-138, mitochondria, endothelial cells

## Abstract

Sepsis is defined as a dysregulated host response leading to organ dysfunction, which may ultimately result in the patient’s death. Mitochondrial dysfunction plays a key role in developing organ dysfunction in sepsis. In this study, we explored the efficacy of the novel mitochondrial protective compound, SUL-138, in sepsis models in HUVECs and mice. In LPS-challenged HUVECs, SUL-138 preserved mitochondrial membrane potential and oxygen consumption and limited mitochondrial oxidative stress, resulting in increased survival at 48 h. Further, SUL-138 dampened the LPS-induced expression of IL-1β, but not of NLRP3, and IL-18 in HUVECs. Sepsis in mice induced by cecal ligation and puncture (CLP) led to a lower mitochondrial membrane potential and increased levels of mitochondrial oxidative stress in the kidney, which SUL-138 limited. In addition, SUL-138 mitigated the CLP-induced increase in kidney dysfunction markers NGAL and urea. It dampened the rise in kidney expression of IL-6, IL-1β, and ICAM-1, but not TNF-α and E-selectin. Yet, SUL-138 limited the increase in plasma levels of IL-6 and TNF-α of CLP mice. These results demonstrate that SUL-138 supports mitochondrial function, resulting in a limitation of systemic inflammation and preservation of kidney function.

## 1. Introduction

Sepsis is a major health problem worldwide with an incidence of around 50 million people. It is the leading cause of death in hospital settings, with an in-hospital mortality rate of 20% [1,2]. Acute kidney injury (AKI) affects up to two-thirds of patients with sepsis admitted to the intensive care unit and is independently linked to higher mortality rates [3,4,5,6]. Sepsis is defined as a dysregulated systemic inflammation that leads to organ failure and finally death of the patient [7]. Mitochondria play a key role in developing organ dysfunction in sepsis [8,9,10]. Patients with sepsis who have a reduced mitochondrial membrane potential have an increased risk of death [10], and reduced ATP concentrations in skeletal muscle biopsies are related to organ failure [9]. Sepsis-induced mitochondrial dysfunction is characterised by the inability of organs to utilize oxygen (i.e., cytopathic hypoxia), which underscores dysfunction of mitochondria as main oxygen consumers [11]. The efficacy of targeting mitochondria to protect organs is supported by studies administering hydrogen sulfide (H_2_S), which improved mitochondrial membrane potential and ATP production after induction of sepsis by cecal ligation and puncture (CLP) in mice [12]. Similarly, MitoTEMPO improved mitochondrial function and survival in CLP mice [13], and MitoQ improved mitochondrial function and organ function in endotoxemic rodents [14,15]. Thus, preserving mitochondrial function seems to hold the key to preventing organ failure and improving the outcome of patients with sepsis.

Current sepsis therapy is limited to antibiotics and supportive care. Novel, targeted treatments to support mitochondrial function are required to prevent organ dysfunction. Until present, efforts to develop new treatments for sepsis by immunomodulation and increasing antioxidant defence have been unsuccessful [16]. To support mitochondrial function, our group developed a new class of compounds derived from 6-chromanol, called SUL compounds. While SUL compounds have intrinsic antioxidant capacity due to their 6-chromanol group, which is also found in alpha-tocopherol (Vitamin E) and Trolox, their therapeutic efficacy roots in their ability to support mitochondrial complexes I and IV of the electron transport chain. Consequently, SUL compounds maintain ATP production and limit mitochondrial oxidative stress in hypothermic cells [17], and maintain mitochondrial mass, ATP levels, and kidney function during hypothermia and rewarming of rats [18]. Given these actions, we hypothesized that SUL compounds preserve mitochondrial function during sepsis, thus preventing organ damage. In this study, we employ the (S)-enantiomer SUL-138, as this compound encompasses a favourable pharmacokinetic profile, and a favourable safety profile indicated by absence of carcinogenicity and genotoxicity [19].

Here, we analysed the action of SUL-138 on mitochondrial and kidney function and the inflammatory response in sepsis models of LPS-treated endothelial cells and sepsis induced by CLP in mice.

## 2. Results

### 2.1. Effects of SUL-138 on Mitochondria and Survival in LPS-Treated Endothelial Cells

**Figure 1:**
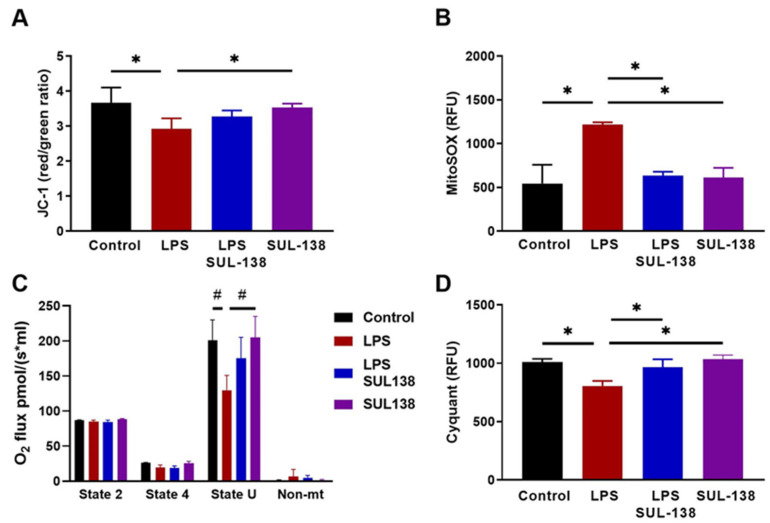
SUL-138 prevents LPS-induced mitochondrial dysfunction and cell death in HUVECs. HUVECs were incubated without and with SUL-138 (10 µM), followed, after 10 min, by incubation with LPS (100 µg/mL). (**A**) LPS for 24 h reduced mitochondrial membrane potential, which was precluded in the presence of SUL-138, as measured by JC-1 fluorescence (n = 16). (**B**) LPS for 24 h increased mitochondrial oxidative stress, which was inhibited by SUL-138, measured by MitoSOX (n = 16). (**C**) LPS for 24 h reduced mitochondrial oxygen consumption in the uncoupled state (State U; CCCP 5 µM), but not in the presence of SUL-138 (n = 4). State 4 was measured in the presence of oligomycin (1 µg/mL) and non-mt in the presence of AMA (5 µM). (**D**) LPS for 48 h reduced cell survival, which was precluded by SUL-138, as measured by Cyquant. (n = 16). Data are represented as mean ± SEM, One-way ANOVA, * *p* < 0.05 or Two-way ANOVA, # *p* < 0.05. AMA, Antimycin A; CCCP, carbonyl cyanide m-chlorophenylhydrazone; CLP, cecal ligation and puncture; LPS, lipopolysaccharide; non-mt, non-mitochondrial.

Endothelial cells form the lining of blood vessels and play a key role in sepsis. Endothelial barrier dysfunction can confer sepsis-induced organ dysfunction by inducing coagulation, immune activation, extracellular fluid accumulation, and loss of organ homeostasis [20,21,22]. To explore the potential protective effects of SUL-138 on mitochondrial function in endothelial cells, human Umbilical Vein Endothelial cells (HUVECs) were treated with LPS for 24 h at 100 µg/ml, with and without treatment by SUL-138. SUL-138 was administered 10 min before LPS. Dose-finding of SUL-138 effects on mitochondrial oxidative stress in the presence of LPS demonstrated an optimal concentration of 10 µM SUL-138 (Figure S1). LPS reduced mitochondrial membrane potential (Figure 1A), increased mitochondrial oxidative stress (Figure 1B), and reduced mitochondrial oxygen consumption (Figure 1C), which were all inhibited in the presence of 10 µM SUL-138 (Figure 1A–C). Endothelial cell death after 48 h of treatment with LPS was reduced by 20% reduction by SUL-138 (Figure 1D). Thus, SUL-138 preserved mitochondrial function in LPS-treated endothelial cells.

### 2.2. Effects of SUL-138 on Inflammation in LPS-Treated Endothelial Cells

**Figure 2:**
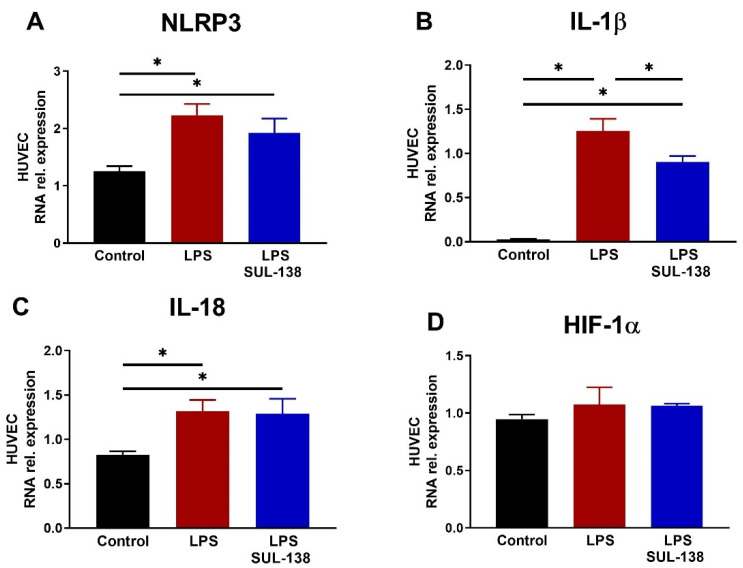
Sul-138 did not affect LPS increased transcription of inflammatory markers in HUVECs. HUVECs were pre-incubated with SUL-138 (10 µM) for 10 min, followed by incubation with LPS (10 µg/mL) for 24 h (n = 8). (**A**) LPS increased NLRP3 RNA expression levels, which was unaffected by SUL-138. (**B**) LPS increased IL-1β RNA expression levels, which was limited in the presence of SUL-138. (**C**) LPS increased IL-18 RNA expression levels, which were similar in the presence of SUL-138. (**D**) HIF-1α RNA expression levels were similar in all groups. Data are represented as mean ± SEM, * *p* < 0.05. HUVECs, human umbilical vein endothelial cells; LPS, lipopolysaccharide.

Since endothelial cells also play an important role in modulating the inflammatory response, we next assessed the effect of SUL-138 on inflammation in LPS-treated HUVECs by measuring mRNA transcript levels. Again, HUVECs were treated with LPS for 24 h at 100 µg/mL with and without treatment of SUL-138 (10 µM). SUL-138 was administered 10 min before LPS. LPS increased mRNA transcript abundance of NLRP3, IL-1β, and IL-18, but did not affect HIF-1α expression (Figure 2A–D). SUL-138 reduced mRNA levels of IL-1β, but did not affect those of NLRP3, IL-18, and HIF-1α (Figure 2A–D). Concluding, SUL-138 only marginally inhibits the inflammatory response in LPS-treated HUVECs.

### 2.3. Effects of SUL-138 on Mitochondrial Function in the Kidney of Septic Mice

**Figure 3:**
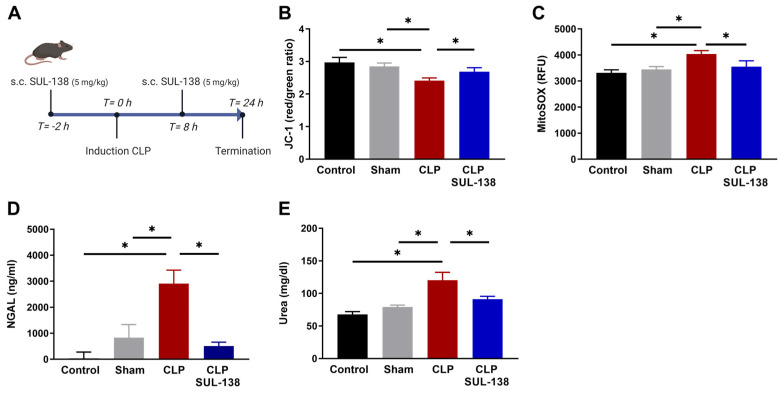
SUL-138 counteracted CLP-induced mitochondrial and kidney dysfunction in mice. (**A**) Timeline of CLP induction. Mice with administration of SUL-138 (5 mg/kg) were injected s.c. 2 h before CLP induction, followed by a second dose at 10 h after CLP induction. After 24 h, CLP plasma was collected, and mitochondria were isolated from the harvested kidneys. (**B**) CLP reduced mitochondrial membrane potential, which was precluded in the presence of SUL-138, measured with JC-1. (**C**) CLP increased mitochondrial oxidative stress, which was inhibited in the presence of SUL-138, measured with MitoSOX. (**D**) CLP increased plasma NGAL levels, which were inhibited in the presence of SUL-138. (**E**) CLP increased plasma urea levels, which were inhibited in the presence of SUL-138. Control (n = 8), sham (n = 8), CLP (n = 8) and CLP SUL-138 (n = 6). Data are represented as mean ± SEM, * *p* < 0.05. CLP, cecal ligation and puncture; NGAL, Neutrophil gelatinase-associated lipocalin.

To study the effects of SUL-138 on mitochondrial and kidney function, and systemic inflammation in mice, we administered SUL-138 (5 mg/kg, injected s.c.) 2 h before induction of sepsis by CLP, followed by a second dose at 10 h after CLP induction. Mice were terminated after 24 h, followed by measurement of mitochondrial and kidney function, and systemic inflammation (Figure 3A). Isolated kidney mitochondria showed a reduced mitochondrial membrane potential (Figure 3B) and increased mitochondrial oxidative stress in septic mice as compared to control and Sham mice (Figure 3C). SUL-138 treatment preserved mitochondrial membrane potential and limited mitochondrial oxidative stress (Figure 3B,C). Kidney function was measured by plasma NGAL and urea levels. SUL-138 inhibited the increase in both NGAL and urea after induction of sepsis by CLP mice (Figure 3D,E). Together, SUL-138 prevented CLP from affecting mitochondrial and kidney function.

### 2.4. Effects of SUL-138 on Renal and Systemic Inflammation of Septic Mice

**Figure 4:**
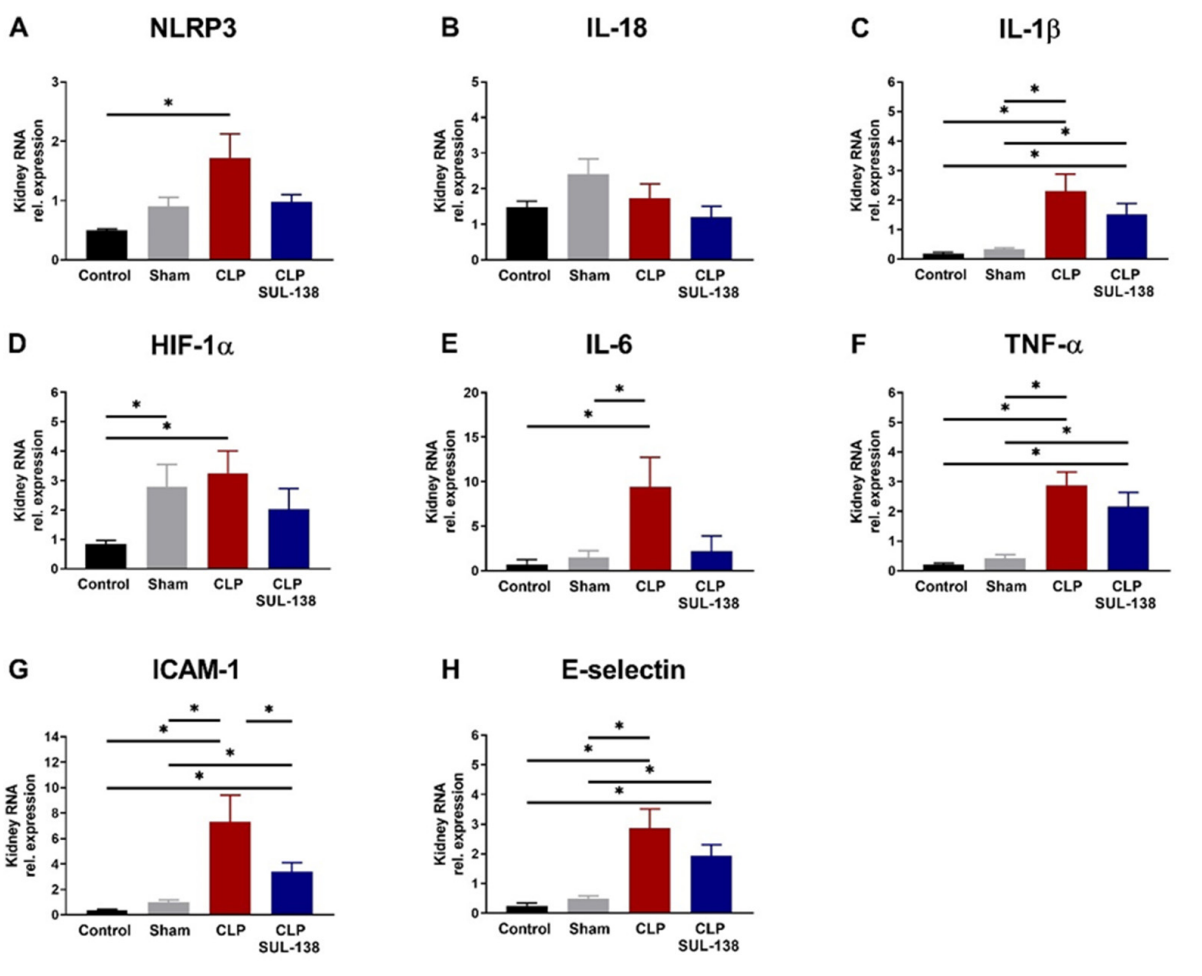
SUL-138 partly inhibited CLP-induced transcription of inflammatory markers in mouse kidney. Mice treated with SUL-138 (5 mg/kg) were injected s.c. 2 h before CLP induction, followed by a second dose at 10 h after CLP induction. Mice were euthanized 24 h after CLP induction and kidneys were collected. (**A**) CLP increased NLRP3 RNA expression levels, which was inhibited in the presence of SUL-138. (**B**) IL-18 RNA expression levels were similar in all groups. (**C**) CLP increased IL-1β RNA expression levels, which was not inhibited in the presence of SUL-138. (**D**) CLP increased HIF-1α RNA expression levels, but not in the presence of SUL-138. (**E**) CLP increased IL-6 RNA expression levels, which was inhibited in the presence of SUL-138. (**F**) CLP increased TNF-α expression levels, which was not inhibited in the presence of SUL-138. (**G**) CLP increased ICAM RNA expression levels, which was inhibited in the presence of SUL-138. (**H**) CLP increased E-selectin RNA expression levels, which was not inhibited in the presence of SUL-138. Control (n = 8), sham (n = 8), CLP (n = 8) and CLP SUL-138 (n = 6). Data are represented as mean ± SEM, * *p* < 0.05. CLP, cecal ligation and puncture.

The effect of SUL-138 on inflammation in the kidney of CLP mice was determined by analysis of mRNA transcript levels of NLRP3, IL-18, IL-1β, HIF-1a, IL-6, TNF-α, ICAM-1, and E-Selectin. CLP increased NLRP3 expression, which was limited in the presence of SUL-138 (Figure 4A). SUL-138 also prevented the CLP-induced increase in IL-1β, IL-6, HIF-1a, and ICAM-1 expression (Figure 4C–E,G), but was without an effect on TNF-α and E-selectin expression (Figure 4F,H). IL-18 expression was unaffected by CLP (Figure 4B). Together, SUL-138 partly limited the inflammatory response induced by CLP but did not fully abolish the inflammatory response in septic mice.

**Figure 5:**
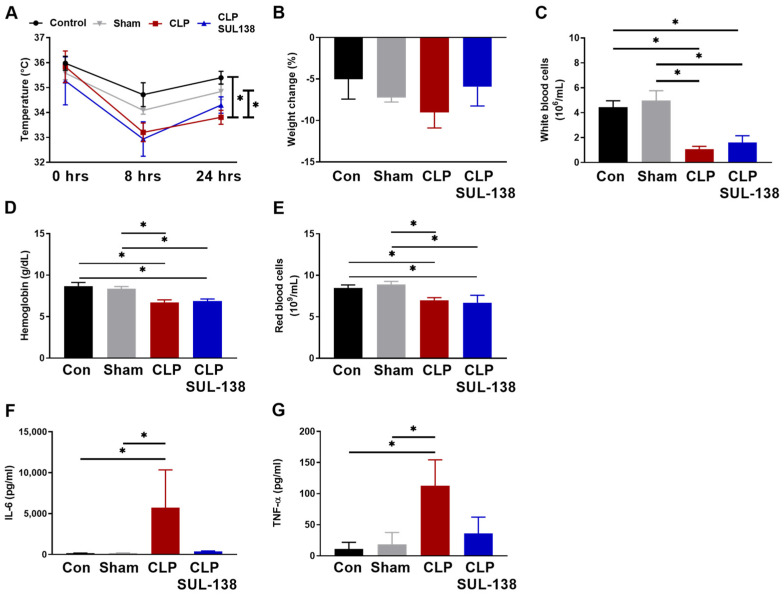
SUL-138 limited the increase in CLP-induced systemic inflammation as evidenced by the normalization of circulating cytokines in mice. Mice treated with SUL-138 (5 mg/kg) were injected s.c. 2 h before induction of CLP, followed by a second dose at 10 h after induction of CLP. Blood samples were drawn 24 h after sepsis induction. (**A**) Xiphoid temperature was reduced in the CLP group, while xiphoid temperature in the CLP SUL-138 group was not significantly reduced after 24 h. (**B**) Body weight was unchanged between groups. (**C**–**E**) CLP reduced white blood cell counts, hemoglobin levels, and red blood cell counts, which were unaffected by SUL-138. (**F**,**G**) CLP increased IL-6 plasma and TNF-α levels, which were precluded by SUL-138. Control (n = 8), sham (n = 8), CLP (n = 8) and CLP SUL-138 (n = 6). Data are represented as mean ± SEM, * *p* < 0.05. CLP, cecal ligation and puncture.

Next, the effects of SUL-138 on systemic inflammation in CLP mice were assessed. SUL-138 limited the CLP-induced reduction in body temperature at 24 h after induction (Figure 5A). Contrarily, SUL-138 did not affect the weight loss of CLP mice (Figure 5B), and the CLP-induced reduction in the number of circulating white and red blood cells, and hemoglobin level (Figure 5C–E). Finally, SUL-138 mitigated the CLP-induced increase in the levels of systemic circulating cytokines IL-6 and TNF-α (Figure 5F,G).

## 3. Discussion

Here, we show protective effects of the novel chromanol-compound SUL-138 on preservation of mitochondrial function in human endothelial cells and murine kidney in sepsis. LPS-induced mitochondrial dysfunction in human endothelial cells as evidenced by a reduced mitochondrial membrane potential and increased mitochondrial oxidative stress levels, together with increased mRNA expression of the inflammation markers NLRP3, IL-18, and IL-1β. Incubation of cells with LPS led to endothelial cell death. Pre-treatment with SUL-138 prevented mitochondrial dysfunction and cell death, and partially dampened the inflammatory response. In mice, CLP results in systemic and renal inflammation, mitochondrial dysfunction, and kidney dysfunction, of which mitochondrial dysfunction and kidney failure were ameliorated by SUL-138 treatment. While SUL-138 partly limited systemic inflammation demonstrated by lowered plasma IL-6 and TNF-α and dampened a decrease in body temperature during sepsis, it did not mitigate the effects of CLP on white blood cell count. In the kidney of CLP mice, SUL-138 partly limited kidney-specific expression of the inflammation markers NLRP3, HIF1-a, IL-6, and ICAM-1, but not IL-1β, TNF-α, and E-selectin. Together, the protective effects of SUL-138 in sepsis originate mainly from the preservation of mitochondrial, endothelial, and sepsis-AKI, rather than primarily abolishing the inflammatory response. 

The LPS-stimulated cell models are widely used for studying inflammation and testing the efficacy of novel compounds. In our model, we used human endothelial cells, since endothelial dysfunction is a hallmark in developing organ dysfunction during sepsis [21,23]. LPS’ action on endothelial cells comprises the activation of an immune response by stimulating the adhesion of leukocytes to the cells through upregulating ICAM-1 and VCAM-1 [24,25], and LPS disrupts mitochondrial function by reducing mitochondrial membrane potential and increasing mitochondrial oxidative stress [24,26], cumulating in endothelial leakage [27]. Studies on mitochondria showed the importance of mitochondrial dysfunction underlying organ dysfunction in sepsis. Skeletal muscle ATP concentrations in biopsies were related to organ failure in patients with sepsis [9], and platelet mitochondrial membrane potential correlates with non-survival [10]. An experimental model of early AKI was not associated with changes in renal blood flow, oxygen delivery, or histological appearance [8]; the inability of organs to utilize oxygen (i.e., cytopathic hypoxia) underscores the dysfunction of mitochondria [11]. Together, studies on mitochondria showed the importance of mitochondria as a potential therapeutic target. 

The kidneys have a relatively high mitochondrial mass to allow effective resorption of solutes by the tubular cells, which is a process that requires a lot of ATP. The CLP mouse model displays AKI, endothelial dysfunction, and important markers of mitochondrial dysfunction, in line with our study, and is the gold standard in sepsis research [28,29,30,31]. The induction of sepsis by a polymicrobial infection and administration of antibiotics increases the model’s face, construct, and predictive validity. Our CLP mouse model contracts AKI reflected by increased urea and NGAL [32,33], in accordance with patients with severe sepsis, of whom more than 50% develop AKI [34]. This makes kidney injury a prominent organ failure in sepsis; preserving mitochondrial function with SUL-138 may ameliorate AKI in patients with sepsis. 

Since clinical trials on modeling inflammation have been unsuccessful in sepsis, the therapeutic focus shifted toward more precise targets, such as mitochondrial-targeted therapies [35,36]. The novel compound SUL-138 demonstrated mitochondrial supportive effects in previous studies [17,18,37], with a specific improvement of the mitochondrial complex I and complex IV [17]. Here we show that treatment with SUL-138 supports mitochondrial function of LPS-stimulated endothelial cells and in the kidney of septic mice. The data are in line with previous observations in CLP mice, where improving cytochrome *c* (complex IV) interactions improved cardiac function by exogenous cytochrome *c* [38]. Further, MitoTEMPO supported kidney complex I activity, improving kidney function [13]. The notion that SUL-138 is primarily acting through the preservation of mitochondrial function is also in line with the absence of actions on the expression of inflammatory markers NLRP3, IL-18, and IL-1β in endothelial cells, and IL-1β, TNF-α, and E-selectin in the kidney. In addition, SUL-138 improved endothelial cell survival during LPS stimulation, and kidney function in sepsis. This follows the role of mitochondria in cell death [39] and findings that suggest supporting mitochondrial function improves cell survival in sepsis [35]. 

Whereas SUL-138 only partly reduced sepsis-associated organ expression of inflammatory cytokines, it lowered sepsis-induced increases in systemic inflammatory markers. This discrepancy may potentially be due to SUL-138 limiting the release of damage-associated molecular patterns (DAMPs) from damaged cells in CLP mice by preserving mitochondrial dysfunction as well as limiting oxidative stress, which are both expected to indirectly lower the inflammatory response induced by sepsis in mice. 

The efficacy of SUL-138 in preserving mitochondrial function, preventing endothelial cell death, and AKI in the pre-clinical models combined with the favourable safety profile from pre-clinical studies make SUL-138 a promising compound to avert organ injury in patients with sepsis [19,37]. However, several limitations have to be taken into account, as well as patient factors that may affect the efficacy and safety of the drug in human sepsis. First, to obtain insight into the efficacy of the drug, as proof-of-principle, we administered the drug before induction of sepsis. An obvious clinical application of SUL-138 would therefore constitute prophylactic treatment in specific procedures, such as major surgery (e.g., abdominal or urogenital surgery with risk of post-operative sepsis) or severely immunocompromised patients. With respect to sepsis patients, further research is required to establish whether early administration of SUL compounds is limiting the progression of disease, in particular preventing end-organ dysfunction. Finally, at present it is unexplored whether SUL will aid in restoring organ failure among patients with severe sepsis in the intensive care unit.

The CLP model is the best available model to study sepsis in terms of face, predictive, and construct validity. By capturing crucial disease features of human sepsis, this model has been widely used to explore mechanisms of organ injury in sepsis and test the potential of therapeutic interventions. While CLP is considered the gold standard pre-clinical model for the study of sepsis, the animals included were healthy young adult male mice. Hence it is uncertain to what extent our results may be translated to females and common classes of sepsis patients, including those at the extremes of ages and those with extensive co-morbidity. Moreover, the nature of the CLP model implies that sepsis was induced by a polymicrobial intra-abdominal infection. Yet, sepsis in humans can arise from different pathogens and infection sites, including infections originating from the lungs, urinary tract, and skin (such as cellulitis or infected wounds). In conclusion, while CLP is a valuable tool for pre-clinical studies of sepsis, it is important to consider the limitations of the model and the variability of sepsis in humans.

## 4. Materials and Methods

### 4.1. Cell Culture

HUVECs were obtained from the RuG/UMCG Endothelial Cell Facility. Briefly, primary isolates of umbilical cords were mixed and subsequently cultured on HUVEC culture medium, which consisted of RPMI 1640 (Lonza, art.nr. BE12-115F, Breda, The Netherlands) supplemented with 20% heat-inactivated fetal calf serum (ThermoFisher Scientific, art.nr. 10082147, Waltham, MA, USA), 2 mM l-glutamine (Life Technologies art.nr. 25030, Carlsbad, CA, USA), 5 U/mL heparin (Leo Pharmaceutical Products, The Netherlands), 1% Penicillin/Streptomycin (Sigma-Aldrich art.nr. P4333, St. Louis, MO, USA), and 50 μg/mL EC growth factor supplement from (Sigma-Aldrich, art.nr. E2759). 

### 4.2. Endothelial Cell Experimental Setup 

Primary HUVECs were cultured in 75-cm^2^ tissue culture flasks (Corning, art.nr. 430720U, St. Louis, MO, USA) at 37 °C under 5% CO_2_/95% air. HUVECs were used for experiments up to passage 8. Experiments were performed in 6-well (Corning art.nr. 3506) or 96-well culture plates (Corning, art.nr. 3596), at 80% confluency. Cells were stimulated with LPS *E. coli* 0111:B4 (Sigma-Aldrich, art.nr. L2630) in different concentrations. Cells were detached with trypsin (Sigma-Aldrich, art.nr. 25300054). All compounds were dissolved in Hanks Balanced Salt Solution (Lonza, art.nr. 10-527F). HUVECs were pre-incubated with SUL-138, which was added 10 min prior to LPS-stimulation. Each experiment was performed in at least triplicate. HUVECs were obtained from the RuG/UMCG Endothelial Cell Facility. Briefly, primary isolates of umbilical cords were mixed and subsequently cultured on HUVEC culture medium, consisting of RPMI 1640 (Lonza, art.nr. BE12-115F, Breda, The Netherlands) supplemented with 20% heat-inactivated fetal calf serum (ThermoFisher Scientific, art.nr. 10082147, Waltham, MA, USA), 2 mM l-glutamine (Life Technologies art.nr. 25030, Carlsbad, CA, USA), 5 U/mL heparin (Leo Pharmaceutical Products, The Netherlands), 1% Penicillin/Streptomycin (Sigma-Aldrich art.nr. P4333, St. Louis, MO, USA), and 50 μg/mL EC growth factor supplement from (Sigma-Aldrich, art.nr. E2759). 

### 4.3. Animal Experimental Setup

All animal experiments were approved by the Institutional Animal Care and Use Committee of the University Medical Center Groningen (IvD nr. 16593). Male C57/BL6J mice were housed at the Central Animal Facility at the UMCG at room temperature at a light-dark cycle of 12:12 h. Animals were fed ad libitum using standard animal lab chow and had free access to drinking water at all times. To induce sepsis, animals were anesthetized by subcutaneous injection of xylazine/ketamine (100/10 mg/kg), followed by administration of buprenorphine (0.1 mg/kg) as analgesic. After confirmation of anesthesia by lack of response to paw pinch and eye reflex, the abdomen was shaved, cleaned, and degermed using a povidone-iodine solution before a 1-cm midline incision was made. The cecum was ligated with a 6-0 suture at half the distance between distal pole and the base of the cecum and punctured once with a 21-Gauge needle (‘through-and-through’ from mesenteric toward anti-mesenteric direction) which is expected to lead to ‘mid-grade’ sepsis [29,30,31]. A small amount of stool (2–3 mm) was then extruded to ensure wound patency. The cecum was repositioned, thereby taking care not to spill fecal material on the wound edges, followed by closure of the abdomen by running sutures to the abdominal musculature (6-0 Safil sutures) and short interrupted sutures to the skin (5-0 Safil), as described by Rittirsch et al. [39]. Next, 1 ml of saline (warmed, 0.9% NaCl, s.c.) was administered to compensate for the expected relative volume depletion due to the onset of sepsis. Following the procedure, mice recovered at 26–28 °C. Broad-spectrum antibiotics (imipenem/cilastatine, 100 mg/kg, s.c.) were administered at 2- and 10-h following surgery, together with analgesics (buprenorphine, 0.1 mg/kg body weight, s.c.). A group of operated animals, in which the cecum was located but not punctured, served as shams. In addition, a group of animals was included that underwent time-matched anesthesia, but no surgery, and served as controls. Mice in the SUL-138 (6-hydroxy-2,5,7,8-tetramethylchroman-2-yl)(piperazin-1-yl)methanone) (Sulfateq bv, Groningen, The Netherlands) treated groups were injected with SUL-138 (dissolved in saline, 5 mg/kg, s.c.) at 2 h before and 8 h after surgery, while mice in the other groups were injected with an equal volume of saline at these time-points. This resulted in an experimental setup of control (n = 8), sham (n = 8), CLP (n = 8) and CLP SUL-138 (n = 6). Mice were sacrificed 24 h after the procedure. Upon euthanization, 40 μL of blood was used to obtain blood cell counts on a Sysmex PoCH 100-iv analyzer hematocytometer. The remainder of the EDTA-anticoagulated blood was separated into plasma by centrifugation at 1600× *g* for 10 min and serum by allowing it to clot for 30 min followed by centrifugation at 3000× *g* for 10 min. Plasma, serum, and organs were snap-frozen in liquid nitrogen for further analysis.

### 4.4. Mitochondria Isolation from the Kidney

Kidneys were rinsed with Ringer’s solution, connective tissue was removed and immediately stored at 4 °C. Subsequently, the kidneys were homogenized in isolation medium A (220 mM mannitol, 70 mM sucrose, 5 mM TES, 0.1 mM EGTA, pH 7.3 with KOH) and centrifuged at 800× *g* (4 °C, 10 min). The supernatant was transferred to a clean centrifuge tube and centrifuged at 7200× *g* (4 °C, 10 min). The supernatant was stored as a cytosolic fraction and the pellet resuspended with medium A. The pellet fraction was centrifuged at 7200× *g* (4 °C, 10 min). Protein concentrations were determined by Bio-rad DC protein assay using a synergy H4 micro plate reader (Bio-Tek Instruments, Inc., Winooski, VT, USA) at absorbance 750 nm.

### 4.5. Mitochondrial Membrane Potential

Membrane potential was measured by using JC-1 (5,5′,6,6′-tetrachloro-1,1′,3,3′-tetraethyl-imidacarbocyanine iodide). Mitochondrial membrane potential was measured after 30 min. Cells and mitochondria were incubated and measured according to manufacturer’s protocol in the Synergy H4 micro plate reader (Bio-Tek) at excitation/emission of 548/574 nm.

### 4.6. Mitochondrial Oxidative Stress

Mitochondrial superoxide generation was measured using MitoSOX (ThermoFisher Scientific, #M36008) in cells or mitochondria according to manufacturer’s protocols. Fluorescent signals obtained with MitoSOX were quantified after 30 min at 37 °C with the Synergy H4 micro plate reader (Bio-Tek) at excitation/emission of 510/580 nm. 

### 4.7. Mitochondrial Oxygen Consumption

Mitochondrial oxygen consumption was measured using the Oroboros O2k respirometer at 37 °C. Cells were trypsinized and resuspended in 1 ml culture media. State 2 (basal respiration) was measured in the presence culture media. State 4 was measured in the presence of oligomycin (1 µg/mL). State U (uncoupled respiration) was measured in the presence of carbonyl cyanide m-chlorophenylhydrazone (CCCP; 5 µM). Non-mitochondrial respiration was measured in the presence of antimycin A (AMA; 5 µM). Oxygen consumption rates were normalized to the number of 3 × 10^6^ cells per ml. The experiment was repeated 4 times and data were analysed with Datlab 5.

### 4.8. Cell Survival

The cytotoxic effects of LPS were quantified by Cyquant NF Cell Proliferation Assay Kit (ThermoFisher Scientific, #C35006) in cells, according to the manufacturer’s protocol. Cyquant binding to nuclear DNA was measured with the Synergy H4 micro plate reader (Bio-Tek) at excitation/emission of 497/520 nm.

### 4.9. Plasma Cytokine Measurement

To precisely quantify the effect of sepsis severity and treatment with SUL-138 on systemic inflammation, we measured levels of TNF-α, IL-6, and IL-12 in plasma using Mouse DuoSet ELISAs (DY410, DY406 and D419, respectively, RnD-Systems), according to the manufacturer’s instructions. Briefly, ELISA plates (DY990, RnD Systems) were coated overnight with the capture antibody diluted in 100 µL PBS. Plates were washed 3 times with wash buffer (0.05% Tween20 in PBS; 137 mM NaCl, 2.7 mM KCl, 8.1 mM Na_2_HPO_4_, 1.5 mM KH_2_PO_4_, pH 7.2–7.4), followed by blocking for 1 h with 300 µL of reagent diluent (1% probumin *w*/*v* in PBS). Washing was repeated and samples were added to the wells. Plasma samples were diluted 10× for TNF-α, and 100× for IL-6 in reagent diluent. After incubation for 2 h at room temperature, plates were washed, followed by adding 100 µL of detection antibody diluted in reagent diluent to each well. Again, plates were left to incubate for 2 h at room temperature, followed by washing. Finally, 100 µL of substrate solution (DY999, RnD Systems) was added and after 20 min incubating in the dark, 50 µL stop solution (2M H_2_SO_4_) was added. The optical density (OD) was measured using a microplate reader set to 450 nm, while readings at 540 nm were subtracted as a correction to increase accuracy.

### 4.10. Statistical Analysis and Data Presentation

Data were analysed using GraphPad Prism 8. Results were expressed as means with standard error of the mean (SEM). Normality was tested using a Shapiro–Wilk test. Differences between groups were assessed using one-way ANOVA tests or two-way ANOVA tests, differences between groups were assessed with a post hoc comparison using Dunnett’s test. Significance was assumed at 2-tailed *p* < 0.05. Figures were produced with GraphPad Prism 8 and graphical overviews with Biorender.com.

## 5. Conclusions

The novel compound SUL-138 was previously found to improve mitochondrial complex I and complex IV activity and limit mitochondrial oxidative stress [17] with beneficial effects in preclinical models of hypothermia/rewarming and Alzheimer’s disease [18,37]. Our data support these findings and add that SUL-138 supports mitochondrial function, cell survival, and organ function in both human endothelial cells and mice models of sepsis, with partially limiting inflammatory signals. Therefore, SUL-138 represents a compound that supports mitochondrial function, endothelial survival, and has the potential to ameliorate AKI in sepsis

## 6. Patents

HR Bouma, RH Henning, GJW Euverink, G Krenning, AC Van Der Graaf. Compounds for treatment of sepsis. Patent application N2025730, 2020.

## Data Availability

The datasets generated during and/or analysed during the current study are available from the corresponding author on reasonable request.

## Supplementary Materials

The following supporting information can be downloaded at: https://www.mdpi.com/article/10.3390/ijms24076330/s1.
