# Supplementary material for: The Novel Compound SUL-138 Counteracts Endothelial Cell and Kidney Dysfunction in Sepsis by Preserving Mitochondrial Function
Source: Int J Mol Sci. 2023 Mar 28;24(7):6330. doi: 10.3390/ijms24076330 (PMC10094718; doi:10.3390/ijms24076330)
Supplement: Supplementary file 1 [file ijms-24-06330-s001.zip › ijms-2267117-supplementary.pdf]

International Journal of Molecular Sciences

Supplementary Materials: The following supporting information can be downloaded at: https://www.mdpi.com/article/10.3390/ijms24076330/s1.

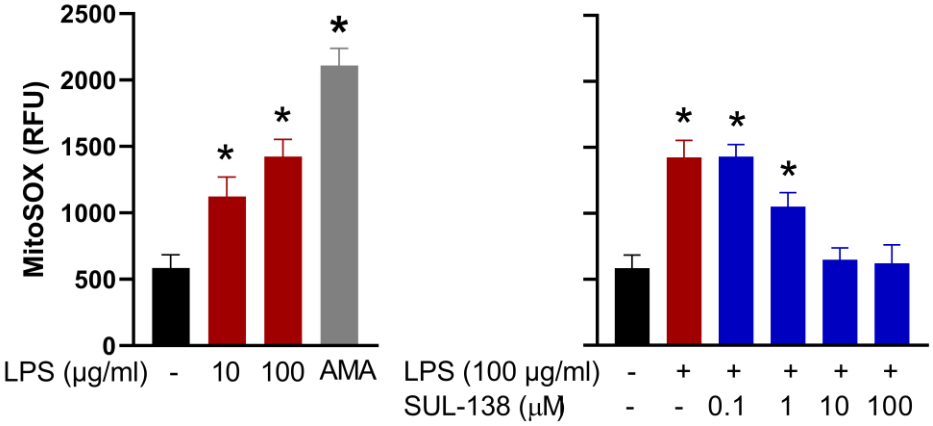

Figure S1: Effect of different SUL-138 concentrations on mitochondrial oxidative stress in HUVECs incubated with LPS. HUVECs were incubated without and with SUL-138, followed after 10 min by incubation with LPS (100 µg/mL). A) LPS for 24 h increased mitochondrial oxidative stress, which was inhibited by SUL-138, measured by MitoSOX (n = 8). Differences between groups were determined with an One-way ANOVA test, and a post hoc analyses compared to control. Data are represented as mean ± SEM, * $P < 0.05$.
